# A Glutaraldehyde-Free Crosslinking Method for the Treatment of Collagen-Based Biomaterials for Clinical Application

**DOI:** 10.3390/bioengineering10111247

**Published:** 2023-10-25

**Authors:** Marvin Steitz, Sabra Zouhair, Mahamuda Badhon Khan, Alexander Breitenstein-Attach, Katharina Fritsch, Sugat Ratna Tuladhar, Dag Wulsten, Willem-Frederik Wolkers, Xiaolin Sun, Yimeng Hao, Jasper Emeis, Hans-E. Lange, Felix Berger, Boris Schmitt

**Affiliations:** 1 Department of Pediatric Cardiology and Congenital Heart Disease, German Heart Center Berlin (Charité), D-13353 Berlin, Germany; 2 Department of Pediatric Cardiology and Congenital Heart Disease, Charité University Medicine Berlin, D-13353 Berlin, Germany; 3 German Centre for Cardiovascular Research, D-10785 Berlin, Germany; 4 Department Dynamics and Transport in Quantum Materials, Helmholtz-Zentrum Berlin für Materialien und Energie GmbH, D-14109 Berlin, Germany; 5 Lower Saxony Centre for Biomedical Engineering, Implant Research and Development, University of Veterinary Medicine Hanover, D-30625 Hannover, Germany; 6 Julius Wolff Institute—Center for Musculoskeletal Biomechanics and Regeneration, D-13353 Berlin, Germany

**Keywords:** biomaterials, pericardium, crosslinking, collagen, glutaraldehyde-free, implantology, biomedical devices, regenerative medicine, tissue application

## Abstract

Biological bioprostheses such as grafts, patches, and heart valves are often derived from biological tissue like the pericardium. These bioprostheses can be of xenogenic, allogeneic, or autologous origin. Irrespective of their origin, all types are pre-treated via crosslinking to render the tissue non-antigenic and mechanically strong or to minimize degradation. The most widely used crosslinking agent is glutaraldehyde. However, glutaraldehyde-treated tissue is prone to calcification, inflammatory degradation, and mechanical injury, and it is incapable of matrix regeneration, leading to structural degeneration over time. In this work, we are investigating an alternative crosslinking method for an intraoperative application. The treated tissue‘s crosslinking degree was evaluated by differential scanning calorimetry. To confirm the findings, a collagenase assay was conducted. Uniaxial tensile testing was used to assess the tissue’s mechanical properties. To support the findings, the treated tissue was visualized using two-photon microscopy. Additionally, fourier transform infrared spectroscopy was performed to study the overall protein secondary structure. Finally, a crosslinking procedure was identified for intraoperative processing. The samples showed a significant increase in thermal and enzymatic stability after treatment compared to the control, with a difference of up to 22.2 °C and 100%, respectively. Also, the tissue showed similar biomechanics to glutaraldehyde-treated tissue, showing greater extensibility, a higher failure strain, and a lower ultimate tensile strength than the control. The significant difference in the structure band ratio after treatment is proof of the introduction of additional crosslinks compared to the untreated control with regard to differences in the amide-I region. The microscopic images support these findings, showing an alteration of the fiber orientation after treatment. For collagen-based biomaterials, such as pericardial tissue, the novel phenolic crosslinking agent proved to be an equivalent alternative to glutaraldehyde regarding tissue characteristics. Although long-term studies must be performed to investigate superiority in terms of longevity and calcification, our novel crosslinking agent can be applied in concentrations of 1.5% or 2.0% for the treatment of biomaterials.

## 1. Introduction

The pericardium is a flask-shaped sac that surrounds the heart and the proximal portions of the great vessels. It exerts mechanical effects, which stabilize the heart in its position and maintain the cardiac geometry and pressure-volume relationships within the cardiac chambers. The pericardium also serves as a physical barrier, protecting the heart from infection and neoplasms. It is classified into the parietal and visceral pericardiums. The parietal pericardium consists of an inner and an outer layer, which merge into adipose tissue. The outer layer, the fibrosa, anchors the heart to the mediastinum. It is made of dense collagen I- and collagen III-bundles with interspersed elastic fibers, while the inner layer, the serosa, is formed by mesothelial cells and is needed for the formation and reabsorption of pericardial fluid [1]. The elastin fibers are arranged among the collagen fibers and are found in all layers. Glycosaminoglycans are uniformly found in both layers of the pericardium, yet they are more abundant in the inner serosal part. The pericardial interstitial cells (PICs) are of mesenchymal origin and appear in two phenotypes: fibroblasts and myofibroblasts. PICs show a spread-out morphology in the serosa and a spindle-shaped morphology in the fibrosa, most probably related to the higher collagen density in the fibrosa [2].

In surgical interventions, collagen-based pericardium is utilized as a biomaterial for repairing or replacing tissue, e.g., for hernia-, tendon-, cardiac-, orbital-, abdominal-, and thoracic wall defect repair, as well as for perivascular patches or heart valve replacement. The utilized pericardial tissue can be of xenogenic, allogeneic, or autologous origin. However, regardless of the tissue‘s origin, it has to be processed before clinical use [3]. Whereas only non-autologous tissue has to obtain treated with antibiotics, cryopreservation, and/or decellularization, all species are getting pre-treated via crosslinking to render the tissue non-antigenic, mechanically strong, or to minimize tissue degradation [4,5]. Crosslinking leads to the introduction of new bonds within the polymer chains of the biomaterial’s extracellular matrix (ECM). The current gold-standard crosslinking agent is glutaraldehyde (GLUT) [6]. Aldehydes are well-established crosslinking agents with a strong affinity for nucleophilic binding sites such as functional amino groups.

**Figure 1:**
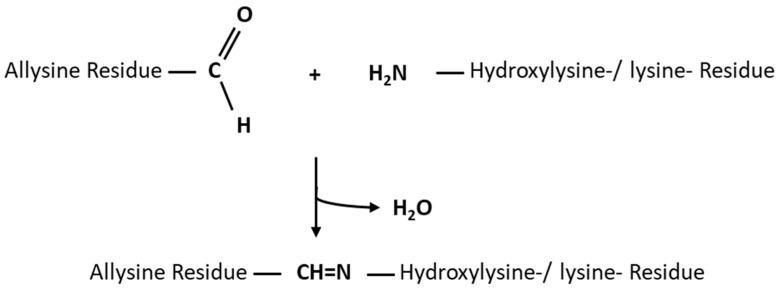
Reaction between the allysine residues and the side chains of hydroxylysine and lysine residues.

Naturally occurring crosslinks within collagen-based tissues are also formed by the reaction of aldehyde groups with allysine, which is highly present in collagen. Therefore, residues of the amino acid lysine are partially hydroxylated, yielding hydroxylysine, which, in turn, obtains enzymatically oxidized by lysyl oxidase, yielding allysin. Subsequently, different crosslinking reactions occur: (i) intramolecular crosslinks are formed by an aldol condensation reaction of two aldehyde groups, and (ii) intermolecular crosslinks are formed by the reaction of the aldehyde group of lysine and the ε-amino group of a hydroxylysine or lysine residue of an adjacent helix (as shown in Figure 1) [7].

**Figure 2:**
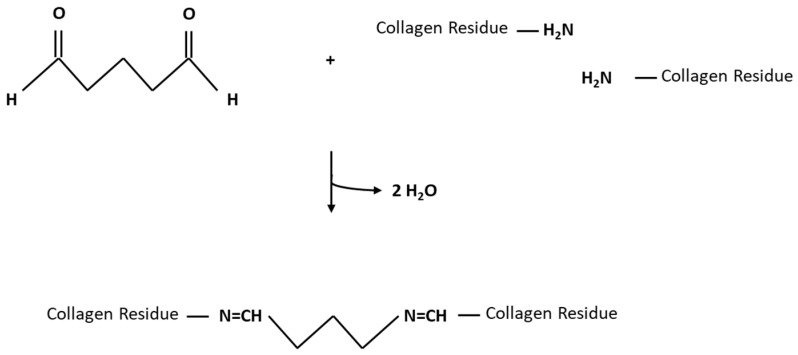
Cross-linking reaction between the aldehyde group of GLUT and the ε-amino group of lysine residues results in a covalent bond.

Although the reaction mechanism of GLUT is not fully elucidated, it was shown that it reacts with the collagen’s amino acids or their functional groups. The ε-amino group of hydroxylysine or lysine reacts with an unsaturated aldehyde sidechain of GLUT upon a nucleophilic attack (as shown in Figure 2) [8].

However, GLUT treatment of tissue compromises its remodeling potential, leading to calcification and resulting in tissue degeneration [5]. This is due to mechanisms: 

Prostheses made from biological tissue are often subjected to dystrophic calcification, which is driven by the deposition of calcium phosphates on cell debris and fibrous components. Since GLUT is an electrophilic compound, it can alter RNA, DNA, and protein synthesis by binding with nucleophiles, leading to cell death. Consequently, cell debris emerges. The cell’s Na^+^/Ca^2+^ exchanger and calcium-dependent ATPase, which maintains a low intracellular calcium level, cease as a result of cell death. This leads to an influx of calcium from the surrounding blood and, subsequently, mineralization. Calcium accumulates at the cell’s phosphate-rich membrane and organelle surfaces and binds with acidic phospholipids and calcium-binding proteins. The resulting microenvironment favors the nucleation of calcium-phosphate crystals (hydroxyapatite), leading to a gradual calcification of the tissue [9].

Tissue is exposed to different mechanical forces, like bending deformation, tension, and shear stress. Because of its chemically altered ECM, GLUT-treated tissue is prone to stress-driven fiber damage leading to mechanical degeneration, which also promotes calcium deposition [9].

It was also believed that GLUT treatment eliminates the immunogenicity of allografts and xenografts. However, current research suggests that the host immune response is not completely suppressed and exogenous tissue is still capable of provoking immune responses [9]. Although standard GLUT fixation protocols eliminate the immunogenicity of protein antigens, immunogenic and xenogenic carbohydrate antigens can persist [10]. These antigens then get detected via direct, semi-direct, and indirect presentation, triggering an immune response [11]. This response may promote degeneration onset via multiple mechanisms. For example, one prominent factor in xenografts leading to calcification is the immune system’s reaction to the carbohydrate Galα(_1,3_)-Galβ(_1-4_)GlcNAc-R (α-Gal) epitope [12,13]. The immune response in primates leads to an interaction of anti-Gal antibodies and the α-Gal epitope [14]. After implantation, the tissue gets actively infiltrated by immune cells that produce proteolytic enzymes, calcium-binding proteins, and reactive oxygen species, leading to degeneration and calcification [9].

In addition to dystrophic calcification, there are several other processes of GLUT-treated biomaterials that can lead to the prostheses’ calcification. GLUT crosslinking of the basic amino acids in collagen helices leads to an impairment of charge balances and, therefore, provides binding sites for the positively charged calcium ions and nucleation sites for calcification [12]. In addition, GLUT treatment does not crosslink proteoglycans and glycosaminoglycans, which mask the calcification-prone areas of collagen called “hole zones”. Therefore, these structures are degraded over time, unmasking further nucleation sites.

Since GLUT only crosslinks collagen fibers, the uncrosslinked elastin is vulnerable to degradation caused by mechanical stress and proteolysis, which leads to mineralization and calcification [9]. Moreover, depolymerization of polymeric GLUT, which can exist in a monomeric dialdehyde but also as a dimer, trimer, and polymer structure in an aqueous solution, can lead to the leaching of highly cytotoxic GLUT into the recipient, leading to recipient-related dystrophic calcification [7,8,9]. 

It was reported that high crosslinking densities are associated with M1 macrophage response and inhibition of M2 macrophage polarization (which get activated for protection against bacteria or viruses and are associated with wound healing or tissue repair, respectively), reduced host cell infiltration, increased pro-inflammatory cytokine expression, fibrosis, and delayed wound healing [15].

Since GLUT treatment leads to long-term failure of the treated tissue, alternative crosslinking methods were assessed to achieve an improved balance between tissue stabilization and durability. Over time, different chemical, physical, and biological crosslinking agents have been assessed [3]. One possible alternative is our novel phenolic crosslinking agent (XOP), whose parental structure has been described as a very potent natural crosslinking molecule.

In our former animal study, XOP was utilized to reshape autologous pericardial tissue into a functional bioprosthesis. For this, the tissue was incubated at a low concentration for 69 h [16]. However, the fabrication of bioprostheses from autologous pericardial tissue is commonly performed intraoperatively (e.g., Ozaki procedure [17], patches [18] or via leaflet reconstruction [19,20]). Therefore, this investigation served to shorten the crosslinking procedure of the tissue for intra-operative processing while maintaining the same degree of crosslinking as in the previous preclinical study. For this dose/incubation-time-finding study, the crosslinking degree was assessed via differential scanning calorimetry (DSC), and the previously used incubation time (XOP 0.05%–69 h) served as the reference group. Afterwards, the chosen dose (and incubation time) were further characterized and compared to GLUT.

Therefore, this investigation also served to assess XOP as an alternative crosslinking agent to GLUT. Apart from the DSC measurements, the treated pericardial tissue was assessed for its crosslinking degree by a collagenase assay. In order to evaluate the tissue’s mechanical performance, uniaxial tensile testing was utilized. The tissue’s overall protein secondary structure was investigated utilizing fourier transform infrared spectroscopy (FTIR). The findings were supported by visualizing the treated tissue by two-photon microscopy.

## 2. Materials and Methods

In order to shorten the formerly utilized incubation time while maintaining the same crosslinking degree and to investigate XOP as an alternative to GLUT, different assessments were conducted. All tests were performed in replicates (*n* ≥ 3) and compared to GLUT and an untreated negative control group. Unless otherwise stated, the utilized reagents were purchased from Sigma-Aldrich (Saint Louis, MO, USA). A list of the utilized materials and devices is shown in Appendix B, in Table A1.

### 2.1. Processing of Tissue

Porcine pericardia (Duroc breed; 115–120 kg) were provided by an abattoir (Fleischerei Lehmann, Trebbin, Germany). Homogenous areas regarding ECM composition and thickness were excised above the left ventricle on-site as described elsewhere [21] and transported in phosphate-buffered solution (PBS). Subsequently, the pericardia were cleaned gently with scalpels and forceps from enveloping fat tissue under sterile conditions (Hera Safe 2020; Thermo Fisher Scientific, Waltham, MA, USA). Afterward, the pericardia were incubated in a 50 mL disinfection solution containing PBS + 4.0% Penicillin-Streptomycin (Pen-strep) (*v*/*v*), and 0.8% Amphotericin B (Ampho B) (*v*/*v*) for 24 h at an orbital shaker at room temperature (RT). The disinfection procedure was repeated after 24 h. The patches were stored in a storage solution containing PBS + 4.0% Pen-Strep (*v*/*v*) and 0.8% Ampho B (*v*/*v*) at 4 °C until usage, for a maximum of 7 days.

### 2.2. Tissue Treatment

The disinfected porcine pericardia were treated with two different crosslinking methods: (i) GLUT or (ii) XOP. Untreated pericardia served as a control group. For the GLUT group, the pericardia were incubated with 0.625% GLUT (Charité-Pharmacy, Berlin, Germany) dissolved in PBS for 24h according to the commercial concentration [22].

In order to identify an intra-operative treatment procedure, the XOP group was incubated with different concentrations (based on previous results), dissolved in PBS to the required concentration (as shown in Table 1), and a dose/incubation-time-finding study was carried out.

GLUT and XOP samples were incubated at RT at 150 rpm on an orbital shaker (New Brunswick Scientific Innova 44, Edison, NJ, USA). The reaction was terminated by removing the crosslinking solution and rinsing the samples with PBS. The samples were immediately processed or stored in the storage solution at 4 °C.

### 2.3. Thermoanalytic Stability Testing (Differential Scanning Calorimetry)

DSC was utilized to investigate the degree of crosslinking for the different concentrations and compared with the reference group in order to identify an appropriate dose/incubation-time for an intra-operative application.

The thermal denaturation temperature of the cross-linked pericardial tissues was assessed using a Differential Scanning Calorimeter (Netzsch DSC 404 F1 Pegasus, NETZSCH-Gerätebau GmbH, Selb, Germany). The protein denaturation peak tends to be complex in biological samples. Therefore, the crosslinking degree of collagen was determined by the more robust endothermic onset temperature (T_onset_), representing the protein denaturation temperature (T_d_), as described elsewhere [23]. The T_onset_ of the reference group served as the desired crosslinking degree. Samples from each group were carefully cut and placed flat in aluminum pans (Mettler Toledo, Columbus, OH, USA), hermetically sealed using a pressure welding machine (NETZSCH-Gerätebau GmbH, Selb, Germany), and a DSC run was performed. Using an empty pan as a reference, the samples were heated from 10 °C to 120 °C at a rate of +10 °C min^−1^ in a synthetic air environment. A pilot run verified the T_d_ range for the GLUT and the control group, which compared well with previous results described elsewhere [24]. Netzsch Proteus thermal analysis software (Netzsch, Selb, Germany) was used for evaluation.

### 2.4. Enzymatic Stability Testing (Collagenase Assay)

Approximately 1 × 1 cm cross-linked- or native control patches underwent collagenase hydrolysis (*n* = 3). The patches were freeze-dried using a lyophilizer (Labconco FreeZone 2.5, Kansas City, MO, USA) and weighed afterwards. Subsequently, each patch was incubated in 0.5 mL of 75 CDU/mL type I collagenase for 12 h at 37 °C, as described elsewhere [25]. Finally, the tissue was again freeze-dried and weighed. Finally, the weight loss ratio [%] was calculated as described elsewhere [26]:

$$\mathrm{Weight}\;\mathrm{loss}\;\mathrm{ratio}=\frac{\mathrm{Weight}\;\mathrm{before}\;\mathrm{hydrolysis}\;-\;\mathrm{Weight}\;\mathrm{after}\;\mathrm{hydrolysis}}{\mathrm{Weight}\;\mathrm{before}\;\mathrm{hydrolysis}}\times \;100$$

### 2.5. Protein Structure (Fourier-Transform Infrared Spectroscopy)

A Perkin-Elmer 100 FTIR spectrometer (PerkinElmer, Norwalk, CT, USA), which featured a triglycine sulfate detector and an attenuated total reflection accessory equipped with a pressure arm and a diamond/ZnSe crystal, was used to assess the secondary protein structure of native or crosslinked pericardium. Tissue patches (*n* = 3; 6 mm in diameter) were collected from all experimental groups and processed following an established procedure as described more detailed elsewhere [27]. Afterwards, the samples were immersed in deuterium oxide for 2 h in order to eliminate interference from H_2_O absorption bands in the protein’s amide-I region. After placing the samples in the spectrometer, their infrared absorption spectra were recorded, employing the following parameters: resolution of 4 cm^−1^, co-adding of 4 interferograms, and a wavenumber range of 4000–900 cm^−1^. The spectral regions of 1700–1600 cm^−1^ were chosen subsequently, and second derivatives were computed using a 13-point smoothing factor (Omnic software; Thermo-Nicolet, Madison, WI, USA) and normalized to resolve differences in peak intensities. Following this, changes in the overall protein secondary structure induced by any of the treatments were assessed using the band intensity ratio, which was calculated using the following formula:

R = Xi/Xj

Xi corresponds to the spectral absorbance value at 1630 cm^−1^, while Xj corresponds to the spectral absorbance value at 1650 cm^−1^.

### 2.6. Two-Photon Microscopy

Images were realized utilizing a Nikon A1R + Multiphoton system (Nikon, Minato City, Tokyo, Japan) equipped with a 25 × 1.1 numerical aperture water-dipping immersion objective. Second harmonic generation (SHG) was utilized to visualize the micro-structure of the collagen fibers with an excitation wavelength of 820 nm and an emission wavelength of 410 nm. On the other hand, two-photon-excited fluorescence signals with an emission filter set at 525/50 nm are used to visualize the elastin fibers.

### 2.7. Biomechanical Characterization (Low Strain Rate Uniaxial Tensile Testing)

Tensile testing was performed using an Instron 3365 dual-column Universal Testing System (Instron, Bucks, UK). Tissue specimens (*n* = 6 per group) were tested under uniaxial tension in the tissue’s circumferential orientation at 37 °C in a temperature-controlled bath filled with PBS solution. The collagen fiber orientation was determined using a self-designed lightbox. Circumferential samples were isolated by cutting strips of 10 mm × 5 mm parallel to the collagen fiber. The average tissue thickness of each sample was determined by measuring the patches at three different points using a digital gauge with an accuracy of ±0.1 mm (Mitutoyo, Andover, UK).

Due to the similar biomechanical behavior of the tissue’s opposite directions, as previously described elsewhere [28], testing was exclusively performed in the circumferential direction. Low-force uniaxial tensile testing was conducted as described more detailed elsewhere [29]: The tested specimens were initially preloaded with a force of approximately 0.1 N and subsequently subjected to loading to failure at a low strain rate of 20 mm/min. Sandpaper was used to prevent the samples from slipping off the clamps.

Throughout the testing process, both the load (F, in Newtons) and the extension (Δl, in millimeters) were continuously measured and then converted into engineering stress (σ, in megapascal (MPa)) and strain (ε, dimensionless). After plotting the stress-strain curve, three biomechanical parameters were calculated and averaged in each group: Young’s modulus of the collagen phase (collagen phase modulus, coll-e) in MPa, failure strain (%), and ultimate tensile strength (UTS), representing the elasticity, maximum strain, and maximum stress of the respective treated samples.

### 2.8. Statistical Analysis

For the statistical analysis, GraphPad Prism (GraphPad Software, San Diego, CA, USA) was utilized. The single variables were expressed as the mean with a standard deviation. The different groups were compared using one-way Analysis of Variance (ANOVA), with significance set at *p* ≤ 0.05.

## 3. Results

### 3.1. Denaturation Profile

DSC was used to identify an appropriate dose/incubation-time for intraoperative processing of pericardial tissue by determining the crosslinking degree after tissue fixation. The scans revealed a typical temperature-dependent endothermic peak related to the absorption of heat accompanying tissue shrinkage and the loss of crosslinking. The peak’s extrapolated peak onset temperature T_onset_ was taken as representing the sample’s denaturation temperature T_d_, showing the tissue’s thermal stability and degree of crosslinking. An example is shown in Appendix B, Figure A3.

**Figure 3:**
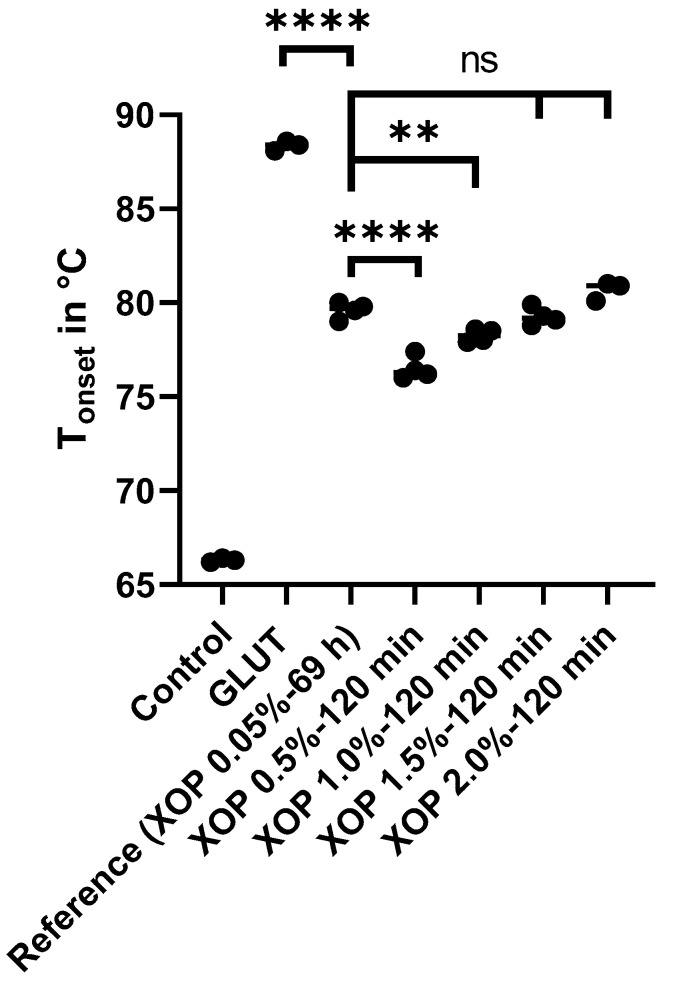
T_onset_ of the control-, GLUT-, or XOP-treated samples. Not significant (ns) = *p* > 0.05. ** = *p* ≤ 0.01. **** = *p* ≤ 0.0001. Standard deviations were calculated within the groups.

For analyzing the kinetics of the treated samples, the T_onset_ was compared between the control, GLUT, and XOP treated groups, including the reference group (XOP 0.05%-69 h). The average T_onset_ for the control, GLUT, and reference groups was 66.2 °C ± 0.1 °C, 88.4 °C ± 0.3 °C, and 79.6 °C ± 0.4 °C, respectively. The average T_onset_ for the XOP 0.5%-120 min, XOP 1.0%-120 min, XOP 1.5%-120 min, and XOP 2.0%-120 min groups was 76.5 °C ± 0.6 °C, 78.3 °C ± 0.4 °C, 79.3 °C ± 0.5 °C, and 80.9 °C ± 0.7 °C, respectively (as shown in Figure 3).

Assuming the T_onset_ of the reference group as the desired crosslinking degree, XOP 1.5%-120 min and XOP 2.0%-120 min, which showed no significant difference regarding their T_onset_, were chosen as the best matching concentration and used for further analysis.

### 3.2. Enzymatic Stability Assessment

**Figure 4:**
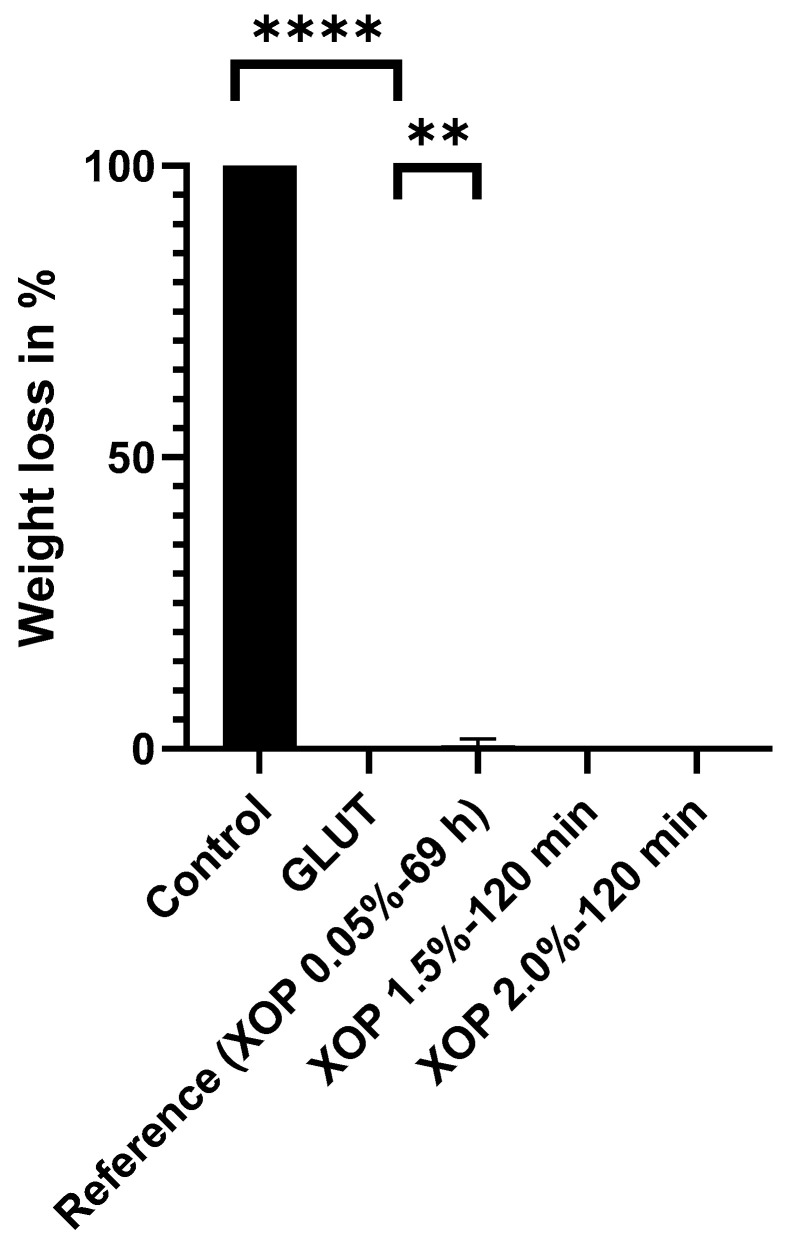
Weight loss of the control-, GLUT-, or XOP-treated samples after challenging them with collagenase. ** = *p* ≤ 0.01. **** = *p* ≤ 0.0001. Standard deviations were calculated within the groups.

For confirmation of the DSC results, the enzymatic stability of the treated samples was assessed by comparing the resistance of collagenase among the different groups. Collagenase is an enzyme found in the body that degrades collagen. The average weight loss of the control, GLUT, and reference groups was 100.00% (completely degraded), 0.00%, and 0.63%, respectively. The weight loss of the XOP-treated samples was 0.00% in all assessed groups (as shown in Figure 4). There were significant differences between the control and all other assessed groups (*p* ≤ 0.0001).

### 3.3. Protein Fingerprint Region Analysis

**Figure 5:**
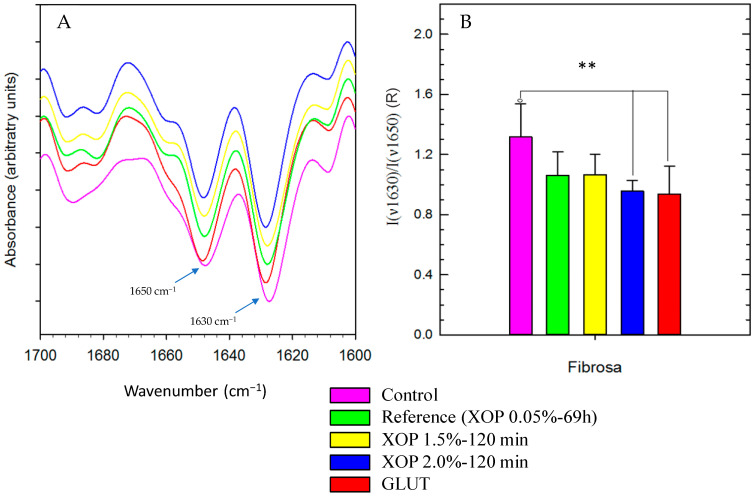
Protein structure analysis of the tissue’s fibrosa of: (**A**) Normalized spectra of the second derivative of the recorded spectra between 1600 and 1700 cm^−1^. (**B**) The ratio of the band intensities of the control-, GLUT-, or XOP-treated samples. The serosa layers did not show significant differences. ** = *p* ≤ 0.01. Standard deviations were calculated within the groups.

For the study of the crosslinking agent’s impact on the overall secondary protein structure, the spectral fingerprint of all groups was determined. From the acquired spectra, the region of interest (1700–1600 cm^−1^) was selected, which includes absorbance bands (C=O stretching and NH bending) resulting from α-helical (~1650 cm^−1^) and β-sheet (~1630 cm^−1^) structures as described elsewhere [30]. The amide-I band region 1700–1600 cm^−1^ is used to describe the correlation between the band shape and the secondary structure contents. Second derivative spectra of native and cross-linked samples were calculated to reveal differences in the amide-I region assigned to the protein secondary structure. The existence of α-helical and β-sheets was confirmed in all groups. There was a slight shift in the treated group’s peaks compared to the control (as shown in Figure 5A). To quantify differences among the groups, the ratio of absorbance values (R) of bands at 1650 and 1630 cm^−1^ was normalized, calculated, and compared among control-, GLUT-, or XOP-treated samples. There was a significant difference between the treated groups and the control group (as shown in Figure 5B), but not within the treated groups, which suggests that GLUT and XOP treatment exert a similar impact on the overall secondary protein structure.

### 3.4. Multi-Photon Microscopy

**Figure 6:**
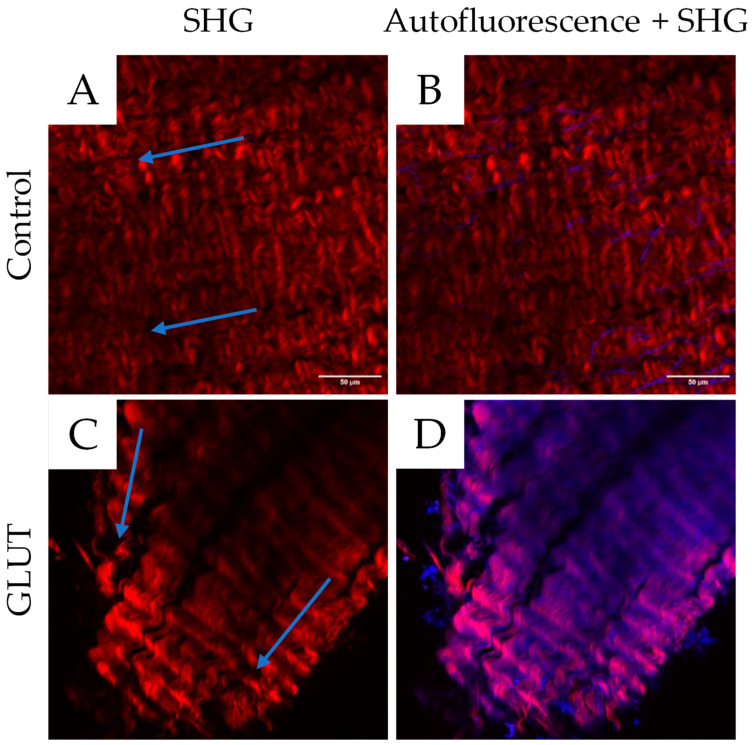

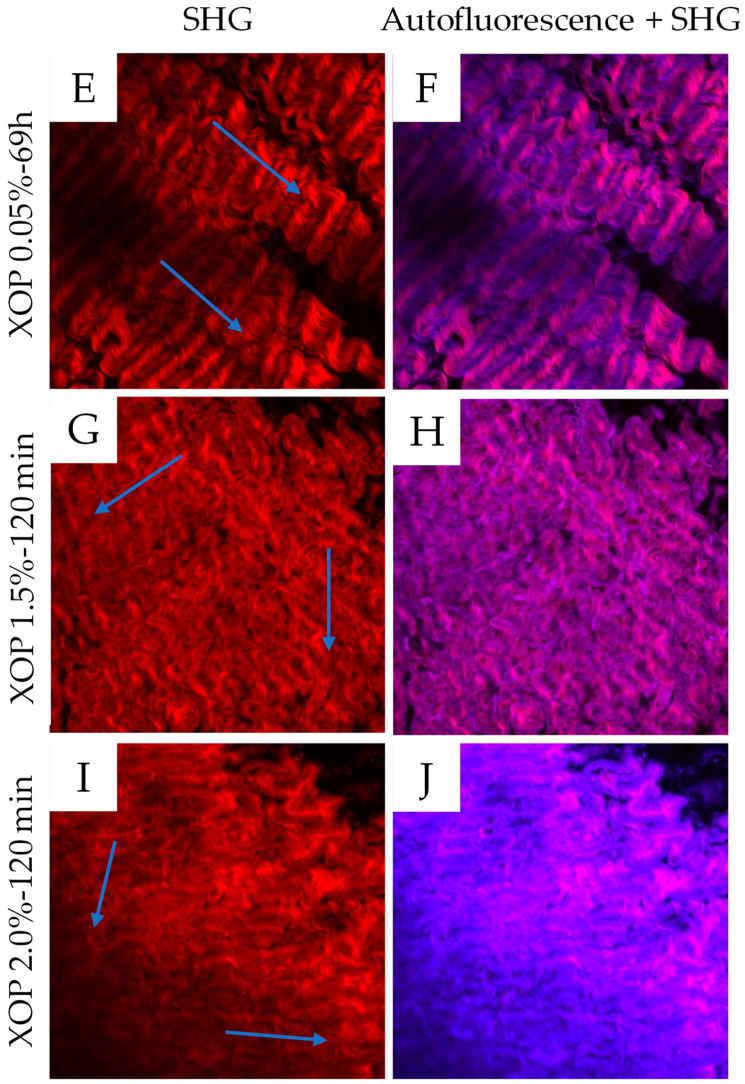
Images (ROIs) of the SHG signal (collagen) (**A**,**C**,**E**,**G**,**I**) and the autofluorescence signal (elastin) + SHG (**B**,**D**,**F**,**H**,**J**) for the control, GLUT, reference, XOP 1.5%-120 min, and XOP 2.0%-120 min, respectively. The SHG channels for collagen (820 nm) and the autofluorescence for elastin (525 nm) are shown in red and blue, respectively. The arrows show the fiber alignment before (Control) and after treatment (GLUT or XOP). The treated samples show an autofluorescence signal of the collagen fibers within the elastin channel, resulting in a purple overlay. Therefore, the elastic fibers are less distinguishable in the treated samples. The scale bar shows a dimension of 50 µm.

The SHG and autofluorescence signals were utilized to visualize collagen and elastin, respectively. After crosslinking, the samples showed an altered collagen fiber distribution, being less aligned in their orientation compared to the control (shown in Figure 6). In the control, a clear collagen fiber alignment is visible. In the GLUT-treated- and reference groups, the fiber alignment is slightly changed. However, the fibers appear less compact than in the control group. The fiber alignment of the XOP 1.5%-120 min and XOP 2.0%-120 min groups is stronger altered after treatment.

The increased background signal in the treated samples within the elastin channel can be explained by the partially overlapping fluorescence ranges of both fibers (collagen and elastin), as explained elsewhere [31]. It was reported that the major source of autofluorescence in collagen is crosslinks, which are increased after treatment with GLUT or XOP [32]. Therefore, an increase in collagen fluorescence, most likely caused by an accumulation of fluorophores within the crosslink sites, results in an increased background signal of the collagen in the elastin channel, as described elsewhere [33].

### 3.5. Biomechanical Characterization of Cross-Linked Tissue

In order to investigate the biomechanical behavior of the cross-linked pericardium, uniaxial tensile testing was conducted. All samples exhibited a typical nonlinear-/exponential-J-shaped stress-strain curve, which is representative of many soft tissues [34]. The biomechanical properties of the control and treated groups are summarized in Table 2.

Table 2 shows the biomechanical properties from the converted stress-strain curves of the control-, GLUT-, or XOP- treated samples. Most groups showed an alteration of all properties after crosslinking. The results primarily show a trend towards an increase in tissue compliance for cross-linked tissue.

**Figure 7:**
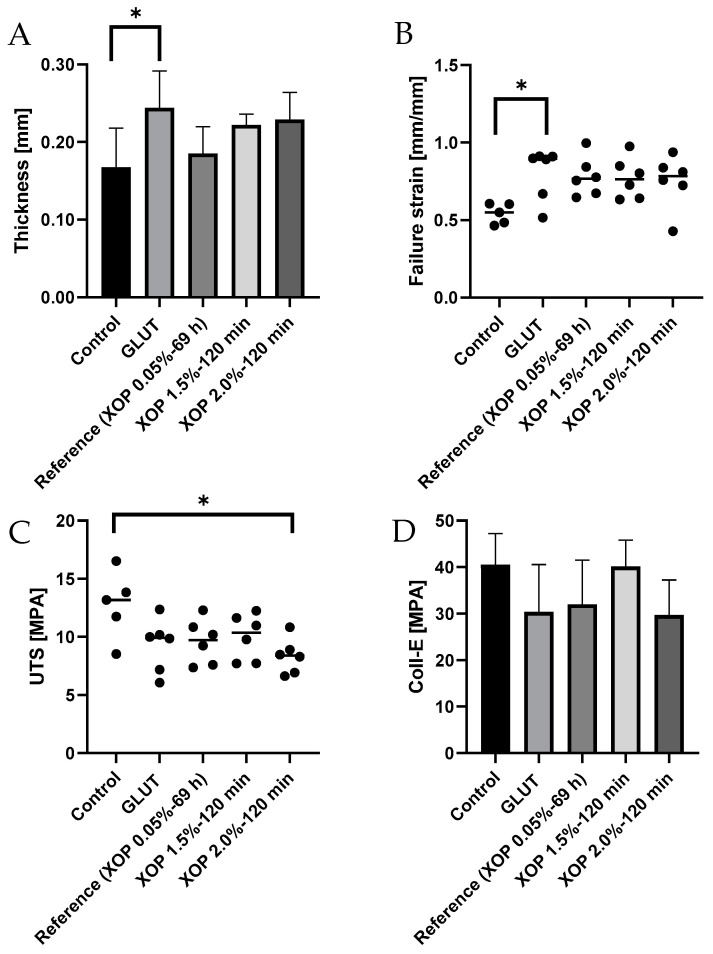
(**A**) thickness, (**B**) failure strain, (**C**) UTS and (**D**) collagen phase modulus of the control-, GLUT-, or XOP-treated samples. * = *p* ≤ 0.05. Standard deviations were calculated within the groups.

There was a tendency towards increasing thickness in the treated groups, showing a significant difference between the control and GLUT groups (*p* < 0.0209) (as shown in Figure 7A).

The failure strain was found to be higher in the cross-linked samples, with a significant difference between the control and GLUT groups (*p* < 0.0421) (as shown in Figure 7B).

The UTS was lower in all treated groups, with a significant difference between the control and the XOP 2.0%-120 min groups (*p* < 0.0172) (as shown in Figure 7C).

A comparable tendency was observed for the collagen-phase modulus (coll-e), correlating with an increase in introduced cross-links, showing tissue with greater extensibility (as shown in Figure 7D). Additionally, the slope in the collagen phase in the treated groups tends to be flatter, with a slightly lower failure point as compared to the control. Also, the elastin phase of the treated groups tends to be broader than the one in the control group.

**Figure A2:**
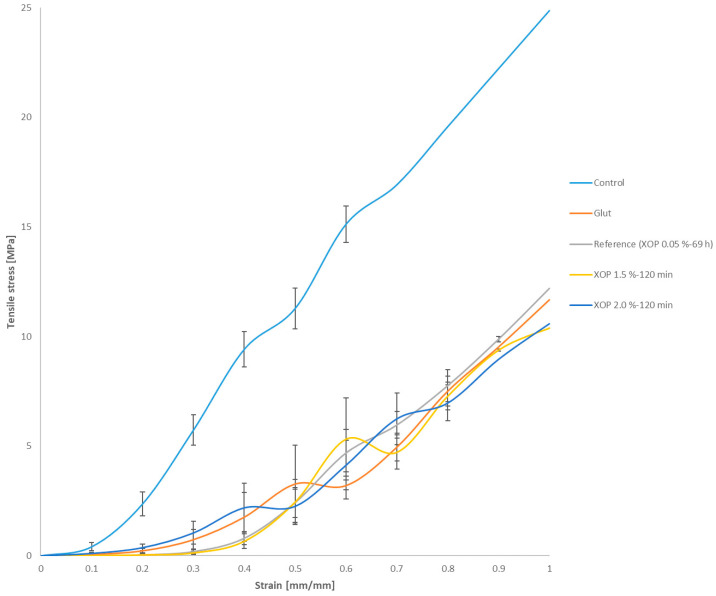
Stress-strain curves of the control, GLUT- and with different concentrations of the control-, GLUT- or XOP-treated samples. Standard deviations were calculated within the groups. For display reasons, the stress-strain curves were plotted without a rupture point.

**Figure A3:**
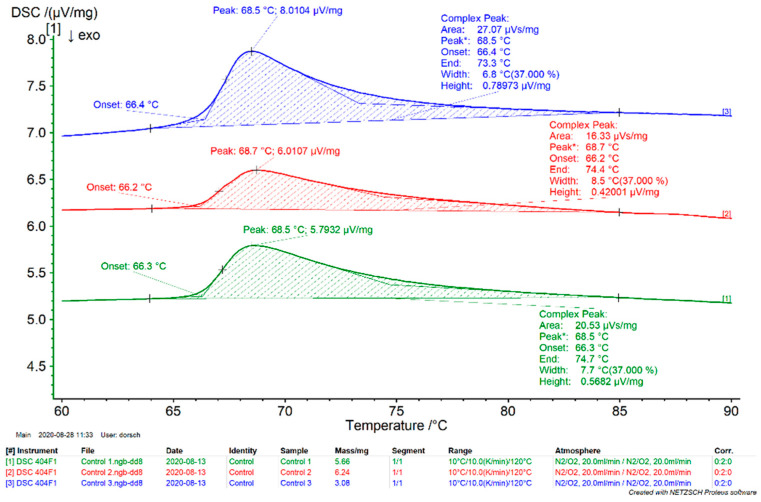
Example of the control group DSC thermogram. The pilot run verified the T_d_ range, comparing it with previous results [24].

The stress-strain curves are shown in the Appendix B, Figure A2.

The scanning electron microscopy assessment is shown in the Appendix A.

## 4. Discussion

In this work, a shortening of the XOP-crosslinking procedure for an intraoperative application has been realized.

Assessments were conducted utilizing porcine pericardium, the use of which is justified as the amount of collagen and fiber organization is similar to human pericardial tissue [35]. 

In order to identify an appropriate dose/incubation-time for the intraoperative processing of biomaterials, DSC was used to analyze the crosslinking degree of different XOP-treated tissues. The crosslinking degree of the animal trial’s incubation treatment (XOP 0.05% for 69 h) served as a reference. The T_d_ defined at the endothermic onset (T_onset_) was used to indicate the crosslinking degree of the pericardial tissues. As described elsewhere, DSC is the prioritized method for the validation of the shrinkage temperature of animal tissue due to its excellent sensitivity and reproducibility, and thus for measuring the extent of crosslinking within a tissue [24]. The slight differences in the assessed T_onset_ within the individual groups can be explained by the intra-species variation of the tissue’s collagen amount [35]. Mimicking the crosslinking degree of the reference concentration was achieved by increasing the concentration while simultaneously decreasing the incubation time. Therefore, the incubation time could be shortened for an intraoperative application (2 h). Furthermore, the significant increase in thermal stability, which was evident for all treated groups, warranted XOP as a crosslinking agent for collagen-based materials. However, the findings show a higher crosslinking degree of GLUT compared to XOP-treated tissue. Before, GLUT had already been described as a crosslinking agent that, compared to other known methods, “[…] gives materials the highest degree of crosslinking […]” [7]. The explanation for this is that aldehydes react with the functional amino groups of collagen and the most effective aldehyde crosslinking agent is a five-carbon molecule, which is the case with GLUT [8].

The two identified concentrations with corresponding incubation times (XOP 1.5%-120 min and XOP 2.0%-120 min), which showed no significant difference from the reference group regarding their crosslinking degree, were further characterized and compared to GLUT.

First, the DSC results were confirmed by the resistance of the cross-linked pericardium against collagenase. Irrespective of the crosslinking method (GLUT or XOP), the samples showed no weight loss after treatment. This resistance of XOP-treated tissue to collagenase also substantiated the suspected reaction between XOP and collagen. The introduction of cross-links within the collagen fibers was also shown by the change in the secondary structure of the tissue’s proteins utilizing FTIR, which is a suitable tool to determine changes in response to tissue treatment [27]. Differences after GLUT or XOP treatment are evident due to changes in the band intensity, which represents the tissues’ structures. Since the protein amide-I band predominantly results from endogenous collagen [27], the observed changes indicate the introduction of cross-links within the collagen fibers. These findings are in line with the biomechanics since the introduction of additional cross-links within the collagen fibers leads to the observed mechanical properties. As described elsewhere [34], the collagen fibers straighten with increasing stress in three phases (elastin-, transition-, and collagen-phase), seen as three sections in the stress-strain curve. The broader elastin phase and the flatter slope in the collagen phase with a lower failure point within the treated groups can be explained by (i) the relaxed collagen fibers in the elastin phase, appearing wavy and crimped, being even stronger crimped in the treated samples, and (ii) the stressed collagen fibers in the collagen phase, appearing straightened and un-crimped, being less aligned and less able to shear with each other in the treated groups. This positive linear trend of the increasing modulus of elasticity in human tissue with an increase in cross-links has been shown before [4]. Similar findings were reported elsewhere [36] and it was concluded that cross-links that were introduced inter-fibrillarly lead to a decrease in the collagen fiber’s ability to shear with each other. To explain this mechanical behavior, a decrease in the collagen’s fibril orientation index was suggested elsewhere [28,37]. It was concluded that this could lead to the generation of a more isotropic network in chemically cross-linked pericardial tissue. It was also observed that crosslinking leads to an increase in fiber crimping [28]. It was suggested that the increase in crimping is due to “[…] the formation of cross-links between the collagen fibers, which is thought to increase the fibril crimp […]” [38]. This in turn could lead to “[…] a larger crimp that may lead to a hidden length [39]. Furthermore, it was reported that GLUT treatment leads to higher rotational stiffness [40]. This increased stiffness and torque resistance could allow the cross-linked tissue to show higher compliance/elasticity in a translational direction. This would explain the higher failure strains in the cross-linked samples. It can therefore be concluded that XOP and GLUT fixation lead to an alteration of the fiber orientation, increased collagen crimping with a resulting “hidden” fiber length, increased rotational stiffness, and a decreased ability of the fibers to shear with each other. These findings are supported by the two-photon microscopy images, which visualize the non-homogenous alignment of the collagen bundles after crosslinking.

All treated samples show a change in thickness after crosslinking. Additionally, the GLUT or XOP cross-linked samples prove to be more compliant, suggesting a larger deformability or reduction in stress relaxation of the tissue compared to the control. It can be concluded that the treatment methods with GLUT or XOP have a similar impact on the mechanical properties of the tissue.

In summary, this study shows that (chemical) crosslinking leads to the reinforcement of existing natural bonds within the collagen-fibers [40] and a resulting change in their alignment due to the introduction of additional crosslinks within the collagen-fibers. These additional crosslinks lead to the observed properties of collagen-based biomaterials:

(i) A higher denaturation or shrinkage temperature of cross-linked tissue is due to the introduction of additional chemical covalent crosslinks, which disrupt at significantly higher temperatures [40] than the fewer existing crosslinks in untreated tissue.
(ii) Increased resistance to collagenase due to the introduction of inter- and intra-molecular crosslinks, leading to a change in the collagen’s protein-structure. Subsequently, enzymatic access to particular cleavage sites is impeded [41].
(iii) The introduction of new crosslinks and the resulting altered fiber alignment also lead to an alteration of the α-helical and β-sheet structures, which was observed in the change of the secondary structure. This also explains an alteration in the fiber alignment observed in the microscopic assessment.
(iv) An increase in compliance and a decrease in ultimate tensile strength are due to the introduction of additional crosslinks, leading to increased rotational stiffness and compression of the fibers and a change in the fiber alignment, resulting in a reduced capacity of the fibers to shear.

It must be noted that in addition to the desired additional crosslinks within the collagen-fibers, further (nucleophilic substitution) reactions between the non-specific reacting chemicals and various structures occur, which also lead to further bonding and changes in the structure.

The assessments chosen in this study have already been described in studies investigating alternative crosslinking methods or characterizing cross-linked tissues and are therefore considered established [23]. The observed results of the thermoanalytic stability [42], enzymatic stability- [43] protein structure- [26] and biomechanical- [36] investigations are comparable to the outcomes of similar studies.

## 5. Conclusions

Pericardial tissue is a promising biomaterial for the replacement or repair of dysfunctional or diseased structures. However, the tissue needs to be pre-treated before use in order to minimize its degradation (and immunogenicity) and improve its mechanical resilience. One crucial treatment step is the crosslinking of the tissue, mostly by means of glutaraldehyde. However, glutaraldehyde treatment leads to calcification and ultimately to tissue degeneration. Therefore, an alternative to glutaraldehyde treatment was assessed in this work. Our novel phenolic crosslinking agent has proven to be an equivalent crosslinking agent to glutaraldehyde. Both fixatives impart tissue with similar characteristics. Our crosslinking agent has the potential to be utilized as a glutaraldehyde-free fixation method. Concentrations of 1.5% or 2.0% could be applied for an intraoperative application.

## 6. Limitations

The investigations carried out give a good initial understanding of the characteristics of XOP-treated tissue. However, they are not sufficient to conclude how the crosslinking treatment with XOP will affect in vivo functional performance. Therefore, further tests for in vitro cytotoxicity, namely contact- and extract assays, should be conducted according to ISO 10993, which describes the biological evaluation of medical devices. Additional in vivo studies would be mandatory to assess the long-term functionality and durability of a pericardial-based prosthesis. In particular, a comparative study should be performed analyzing XOP and GLUT for longevity and calcification.

## 7. Patents

A patent for treating collagen-based biological material with the described crosslinking method is currently pending.

## Figures and Tables

**Table 1:** Different XOP concentrations utilized to treat the tissue samples.

XOP-Concentration	XOP 0.05%	XOP 0.5%	XOP 1.0%	XOP 1.5%	XOP 2.0%
Incubation-Time	69 h	120 min	120 min	120 min	120 min

**Table 2:** Biomechanical properties after treatment.

Biomechanical Properties	Control	GLUT	XOP 0.05%-69h	XOP 1.5%-120 min	XOP 2.0%-120 min
Thickness in mm	0.17 ± 0.05	0.24 ± 0.05	0.19 ± 0.04	0.22 ± 0.01	0.23 ± 0.04
Failure Strain in mm/mm	0.54 ± 0.07	0.80 ± 0.17	0.78 ± 0.13	0.77 ± 0.13	0.75 ± 0.17
UTS in MPa	12.76 ± 2.93	9.27 ± 2.28	9.59 ± 1.91	10.00 ± 1.95	8.34 ± 1.51
Coll-e in MPa	40.54 ± 6.62	30.36 ± 10.19	31.96 ± 9.58	40.16 ± 5.61	29.67 ± 7.54

**Figure A1:**
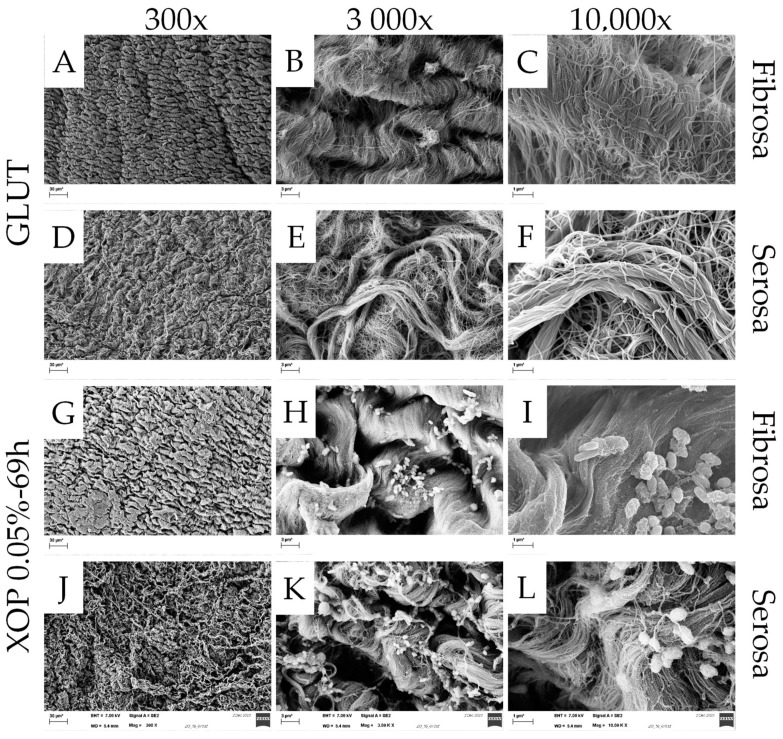

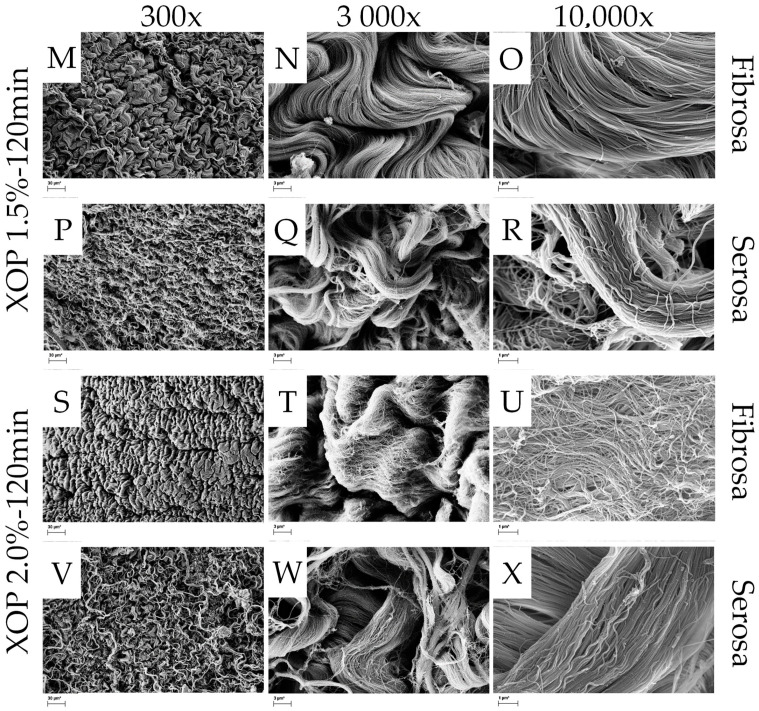
SEM images (representatives of interest) of the serosa (**A**–**C**,**G**–**I**,**M**–**O**,**S**–**U**) or the fibrosa (**D**–**F**,**J**–**L**,**P**–**R**,**V**–**X**), for GLUT, reference, XOP 1.5%-120 min and XOP 2.0%-120 min, respectively. The images in the left-, middle-, and right columns were realized with 300×, 3000×, and 10,000× magnification, showing a scale bar with dimensions of 30 µm, 3 µm, and 1 µm, respectively.

## Data Availability

Not applicable.

## Abbreviations/Nomenclature

**Abbreviation/Nomenclature**	**Meaning**
α-Gal	Galα1,3-Galβ1-4GlcNAc-R
Ampho B	Amphotericin B
Coll-e	Collagen Phase Modulus
DSC	Differential Scanning Calorimetry
ECM	Extracellular Matrix
FTIR	Fourier Transform Infrared Spectroscopy
GLUT	Glutaraldehyde
MPa	Megapascal
PBS	Phosphate-Buffered Solution
Pen-Strep	Penicillin-Streptomycin
PICs	Pericardial Interstitial Cells
R	Ratio of Absorbance Value
RT	Room Temperature
SHG	Second Harmonic Generation
T_d_	Denaturation Temperature
T_onset_	Endothermic Onset Temperature
UTS	Ultimate Tensile Strength
XOP	Novel phenolic crosslinking agent

## Appendix A

### Appendix A.1. Scanning Electron Microscopy

#### Appendix A.1.1. Materials/Method

Surface morphologies were assessed in-house using a GeminiSEM 300 microscope (Carl Zeiss, Oberkochen, Germany). After GLUT- and XOP-crosslinking, postfixation was carried out with 2% OsO_4_ for 2 h, followed by dehydration in an increasing ethanol series, and finally in hexamethyldisilazane. Before analysis, the samples were subjected to gold-palladium sputtering (Sputter coater MED 020, Balzer, Bingen, Germany). Image acquisition was carried out at six magnifications: 300×, 500×, 1000×, 3000×, 5000× and 10,000×. Since SEM analysis requires a fixation step, an untreated control group could not be assessed. All images were realized using a 25 × 1.1 numerical aperture water dipping immersion objective.

#### Appendix A.1.2. Results

Scanning Electron Microscopy was used to visualize the structure of GLUT and XOP-treated pericardial tissue. Wavy collagen bundles were present in the serosal- and fibrosal- layers. The bundles appear less aligned in the serosal layer in all treated groups (as shown in Figure A1). Due to the processing procedure of SEM images, which includes a fixation step, no control group could be evaluated.

## Appendix B

**Table A1:** Utilized materials and devices.

	Materials	Devices
Tissue Treatment	Phosphate-buffered solution (Sigma-Aldrich, Saint Louis, MO, USA) Penicillin-Streptomycin (Sigma-Aldrich, Saint Louis, MO, USA) Amphotericin B (Sigma-Aldrich, Saint Louis, MO, USA) Glutaraldehyde (Charité-Pharmacy, Berlin, Germany)	New Brunswick Scientific innova 44 (Edison, NJ, USA) Hera Safe 2020 (Thermo Fisher Scientific, Waltham, MA, USA)
Differential scanning calorimetry	Aluminum pans (Mettler Toledo, Columbus, OH, USA)	Netzsch DSC 404 F1 Pegasus (F1 Pegasus, NETZSCH-Gerätebau GmbH, Selb, Germany) Netzsch pressure welding machine (NETZSCH-Gerätebau GmbH, Selb, Germany)
Collagenase assay	Type I collagenase (Sigma-Aldrich, Saint Louis, MO, USA) Hank’s Balanced Salt solution (Sigma-Aldrich, Saint Louis, MO, USA) Sodium bicarbonate (Sigma-Aldrich, Saint Louis, MO, USA) Calcium chloride (Sigma-Aldrich, Saint Louis, MO, USA)	Labconco FreeZone 2.5 Lyophilizer (Kansas City, MO, USA)
Fourier-transform-infrared-spectroscopy	Deuterium oxide (Sigma-Aldrich, Saint Louis, MO, USA)	Perkin-Elmer spectrometer (Norwalk, CT, USA)
Two-Photon microscopy		Nikon A1R + Nikon Multiphoton system (Nikon, Minato City, Tokyo, Japan)
Uniaxial tensile loading to failure testing		Instron^®^ 3365 tensile machine (Instron, Bucks, UK) Electronic caliper Mitutoyo, Andover (Mitutoyo, Andover, UK)
Scanning electron microscope	Osmium (IV)-oxid (Sigma-Aldrich, Saint Louis, MO, USA) Ethanol (Sigma-Aldrich, Saint Louis, MO, USA) Hexamethyldisilazane (Sigma-Aldrich, Saint Louis, MO, USA)	GeminiSEM 300 microscope (Carl Zeiss, Oberkochen, Germany)
